# Apigenin-7-Glycoside Prevents LPS-Induced Acute Lung Injury via Downregulation of Oxidative Enzyme Expression and Protein Activation through Inhibition of MAPK Phosphorylation

**DOI:** 10.3390/ijms16011736

**Published:** 2015-01-13

**Authors:** Kun-Cheng Li, Yu-Ling Ho, Wen-Tsong Hsieh, Shyh-Shyun Huang, Yuan-Shiun Chang, Guan-Jhong Huang

**Affiliations:** 1 Department of Chinese Pharmaceutical Sciences and Chinese Medicine Resources, College of Pharmacy, China Medical University, Taichung 404, Taiwan; E-Mail: u9852852@cmu.edu.tw; 2 Department of Nursing, Hungkuang University, Taichung 433, Taiwan; E-Mail: elaine@sunrise.hk.edu.tw; 3 Department of Pharmacology, School of Medicine, China Medical University, Taichung 404, Taiwan; E-Mail: wthsieh@mail.cmu.edu.tw; 4 School of Pharmacy, China Medical University, Taichung 404, Taiwan; E-Mail: sshuang@mail.cmu.edu.tw; 5 Chinese Crude Drug Pharmacy, China Medical University Hospital, Taichung 404, Taiwan

**Keywords:** acute lung injury, apigenin-7-glycoside, lipopolysaccharide (LPS), AOEs (antioxidative enzymes), HO-1 (heme oxygenase-1), MAPK

## Abstract

Apigenin-7-glycoside (AP7Glu) with multiple biological activities is a flavonoid that is currently prescribed to treat inflammatory diseases such as upper respiratory infections. Recently, several studies have shown that its anti-inflammatory activities have been strongly linked to the inhibition of secretion of pro-inflammatory proteins, such as inducible nitric oxide synthase (iNOs) and cyclooxygenase-2 (COX-2) induced through phosphorylation nuclear factor-κB (NF-κB) and mitogen-activated protein kinases (MAPK) pathways. Additionally, inflammation, which can decrease the activities of antioxidative enzymes (AOEs) is also observed in these studies. At the same time, flavonoids are reported to promote the activities of heme oxygenase-1 (HO-1) decreased by LPS. The purpose of this study was to assess these theories in a series of experiments on the suppressive effects of AP7Glu based on LPS-induced nitric oxide production in RAW264.7 macrophages *in vitro* and acute lung injury in mice *in vivo*. After six hours of lipopolysaccharide (LPS) stimulation, pulmonary pathological, myeloperoxidase (MPO) activity, total polymorphonuclear leukocytes (PMN) cells, cytokines in bronchoalveolar lavage fluid (BALF) and AOEs, are all affected and changed. Meanwhile, our data revealed that AP7Glu not only did significantly inhibit the LPS-enhanced inflammatory activity in lung, but also exhibited anti-inflammatory effect through the MAPK and inhibitor NF-κB (IκB) pathways.

## 1. Introduction

Acute lung injury (ALI) and acute respiratory distress syndrome (ARDS) are diseases induced by many extreme conditions including severe sepsis, severe bacterial pneumonia, trauma and burn. The main symptoms of ALI are alveolar edema and uncontrolled neutrophil migrate to the lung [1]. However, no specific therapies are available, and the treatment of this disease has yet to be improved by pharmacologic treatments. Thus, the identification of new molecules that can modulate ALI-associated inflammation is an expected and significant goal of pharmaceutical companies [2].

The fairly uncontrolled pathophysiological mechanisms of ARDS and ALI results in oxidative damage to functional macromolecules such as proteins and lipids, that leads to increasing the thickness of the alveolar wall and pulmonary inflammation. Finally, those symptoms will cause many further disorders, like inflammation, cancer, atherosclerosis, neurodegeneration and aging-related diseases [3]. Pulmonary inflammation is characterized by the upregulation of alveolar capillary permeability, polymorphonuclear neutrophil (PMN) infiltration, secretion of pro-inflammatory cytokines, and transcription factors [3,4]. Lipopolysaccharide (LPS) is derived from the gram-negative bacteria cell wall, not only used to cause ALI in several animal models [5,6,7], but also the most potent bioactivator of the immunological system. Especially in innate immunity, LPS activates the airway epithelial cells and alveolar macrophages to release numerous inflammatory mediators, such as nitric oxide (NO), superoxide anion, cyclooxygenase-2 (COX-2), tumor necrosis factor-α (TNF-α), interleukin-1β (IL-1β) and interleukin-6 (IL-6). Expression of pro-inflammatory cytokines is attributed to the transcription factor being activated by nuclear factor (NF-κB) and three mitogen-activated protein kinase (MAPK) pathways [8,9]. Over-production of these inflammatory mediators is involved in damage of the alveolar capillary barrier and an increase in permeability, which results in much inflammation associated disorders. Therefore, in order to treat inflammatory diseases, the identification of molecules that can modulate ALI-associated inflammation seems to be a good approach by adjusting superoxide anion (O_2_•^−^) and its toxic metabolites, such as hydrogen peroxide (H_2_O_2_), hydroxyl radical, and hypochlorous acid (HClO). In addition, some studies have demonstrated that the antioxidative enzymes (AOEs) including superoxide dismutase (SOD), catalase, and glutathione peroxidase (GPx) can protect tissue against oxidative damage [10].

Recent evidence has suggested that certain compounds are effective in inhibiting PMNs function and infiltration is desirable for the treatment of patients with ALI [11,12]. Natural products like flavone are the most common sources of these drugs. Besides, many studies have further indicated that flavonoids in herbs possess anti-inflammatory activities via interfering with the initiation, development, and progression of inflammatory mediators such as IL-6, TNF-α, and IL-1β in several cell lines through MAPK signaling pathway [13,14].

AP7Glu is a natural flavonoid found in *Lobelia chinesis*, *Teucrium* *gnaphalodes* and dandelion tea, and is stable and has better solubility compared to other flavonoid such as apigenin, although they both have the similar anti-inflammatory capacity [15,16]. There have been reports to demonstrate that AP7Glu has anti-inflammatory effect [9,10,17], but no research on LPS-induced acute lung injury. In the present study, we investigated the tissue protein expression in LPS-stimulated ALI, and examined whether AP7Glu has inhibitory effects through upregulation pro-inflammatory cytokine expressions (IL-1β, IL-6, and TNF) induced by LPS. Furthermore, this study also examined whether IκB, ERK/MAPK, p38/MAPK and JNK/MAPK pathways and oxidative enzymes (AOEs and HO-1) were involved in the mechanisms to underlie the beneficial effects. Therefore, our study was designed to investigate the value of AP7Glu on various aspects of LPS-induced inflammation both *in vivo* and *in vitro*.

## 2. Results

### 2.1. Cytotoxicity

**Figure 1:**
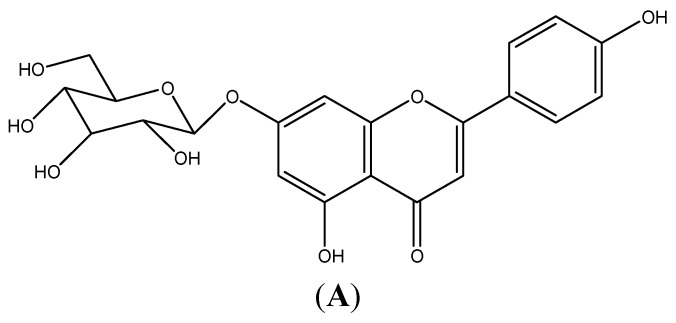

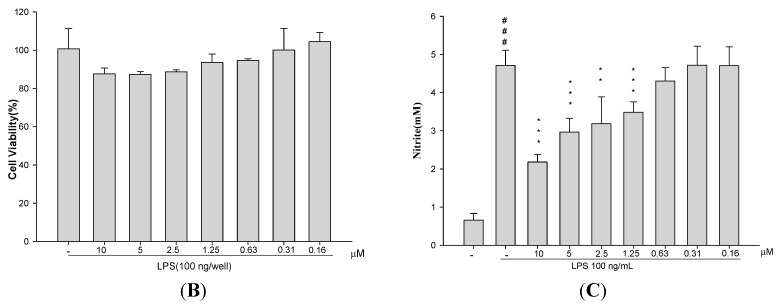
AP7Glu inhibited lipopolysaccharide (LPS)-induced cell inflammation in RAW 264.7 cells. Raw cells were pre-treated with different concentrations of AP7Glu from 10, 5, 2.5, 1.25, 0.63, 0.31, 0.16 μM, or 0 μM (referred as (−)) for 1 h prior to the addition of 100 ng/mL LPS for 24 h. (**A**) The structure of AP7Glu is shown; (**B**) the percentage of cell viability was determined by ELISA; (**C**) The supernatants were harvested and NO production was quantified using ELISA. The data were presented as mean ± SD for the three different experiments performed in triplicate. ^###^ compared with sample of control group (one-way ANOVA followed by Scheffe’s multiple range tests). ** *p* < 0.01, and *** *p* < 0.001 were compared with LPS-alone group.

In order to investigate whether AP7Glu induces cytotoxicity and produces NO in macrophages. Raw 264.7 cells were treated with 0.16–10 μM AP7Glu. LPS was added one hour after incubation. The result in Figure 1B shows that there was no significant difference in the cell viability between LPS with AP7Glu groups and LPS control group. It further indicates that LPS does not induce cell death, and the percentage of cytotoxicity inducing by AP7Glu within the range of 0.16–10 μM is lower than 20%. Moreover, in a dose-dependent manner with an IC_50_ of 9.93 ± 1.32 for Figure 1C, an opposite trend is shown of a decrease in the secretion of NO in LPS-stimulated Raw 264.7 cells when the concentration of AP7Glu is increased in the sample. As a result, in all subsequent cellular and animal experiments, only doses below or equal to 10 μM are applied.

### 2.2. AP7Glu Attenuates Pulmonary Inflammation in LPS-Induced Acute Lung Injury (ALI)

**Figure 2:**
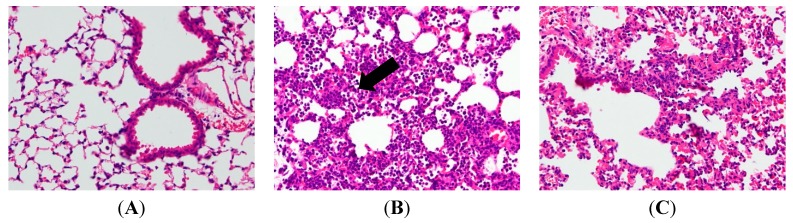

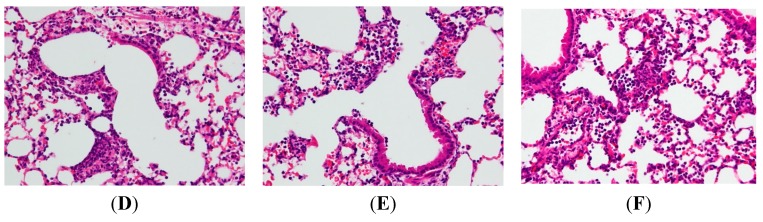
AP7Glu attenuated pulmonary inflammation *in vivo*. Seventy-two hours after LPS injection with or without AP7Glu pretreatments, mice were exsanguinated and their left lower lungs were fixed. Then, tissue sections were stained with hematoxylin and eosin (H&E). The figure demonstrates a representative view (×200) from each group; each bar represents the mean ± SD of 6 mice. (**A**) Control; (**B**) LPS; (**C**) LPS + Dex; (**D**) LPS + AP7Glu-H; (**E**) LPS + AP7Glu-M; (**F**) LPS + AP7Glu-L. The infiltrating neutrophils were more abundant in (**B**) LPS group as shown by arrows.

In an attempt to assess the pathological changes, HE staining was used in our study. After the sacrifice of mice, lung tissue sections were soaked in formalin for two days before histological evaluation. Figure 2B reveals the results in a series of images of notable inflammatory neutrophil infiltration, interstitial edema, interalveolar septal thickening, and intraalveolar and interstitial patchy hemorrhage. However, after treatments of AP7Glu and Dex, the pathological changes in lung tissues are relieved (Figure 2C–E). In contrast to the LPS only group, no evidence of lung injury is found in the normal control group (Figure 2A).

### 2.3. AP7Glu Attenuates Pulmonary Edema in LPS-Induced Acute Lung Injury (ALI)

**Figure 3:**
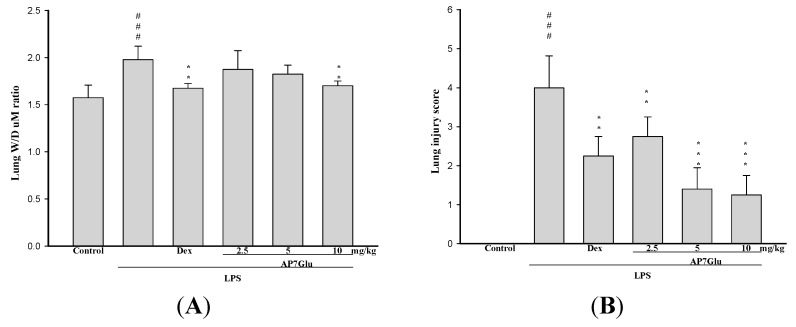
AP7Glu improved pulmonary edema *in vivo*. Seventy-two hours after LPS injection with or without AP7Glu pretreatments, mice were exsanguinated and their right lower lungs were obtained. (**A**) The right lower lungs were used to assess wet to dry (W/D) ratio of lung; (**B**) Severity of lung injury was analyzed by the lung injury scoring system. Each value represents as mean ± SD of 6 mice. ^###^ compared with sample of control group. ** *p* < 0.01, and *** *p* < 0.001 were compared with LPS-alone group.

As for the assessment of non-cardiogenic pulmonary edema, the ratio of the wet to dry weight (W/D) is another critical feature of ALI/ARDS. As shown in Figure 3A, the ameliorations in pulmonary edema are revealed in a dose-dependent manner. In detail, W/D ratio in the LPS group shows a remarkable difference compared with that in the control group. Otherwise, in Figure 3B, the lung injury score stands at around 4 points after LPS injection that is absolutely much higher than that in the other three groups with different amounts of AP7Glu, approximately the highest value only at 3 points. Meanwhile, it also presents a markedly downward trend in the lung injury score with gradually increases in the amount of AP7Glu intraperitoneal injection.

### 2.4. AP7Glu Reduces Cellular Counts and Proteins in BALF

**Figure 4:**
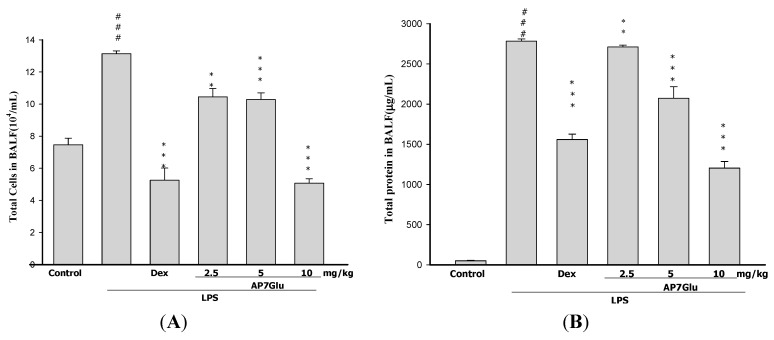
AP7Glu reduced cellular counts (**A**); and total protein (**B**) in BALF. Six hours after LPS injection with or without AP7Glu pretreatments, mice were sacrificed and their lungs were lavaged. Cells in the BALF were collected and cytospin preparations were made. Total cells, and total proteins in BALF were analyzed. Each value represents as mean ± SD of 6 mice. ^###^ compared with sample of control group. (One-way ANOVA followed by Scheffe’s multiple range test). ** *p* < 0.01, and *** *p* < 0.001, were compared with LPS-alone group.

To further identify the anti-inflammatory property of AP7Glu, an important feature is calculating the cellular counts and proteins of PMNs infiltration into the lungs (Figure 4A,B). We evaluated the alterations in leukocyte infiltration occurring in the lungs of LPS-administered mice with or without AP7Glu pretreatment. The number of infiltrating leukocytes was counted by cytometry, and total protein was calculated by Bradford assay on BALF.

### 2.5. AP7Glu Downregulates TNF-α, IL-6, and IL-1β in BALF

**Figure 5:**
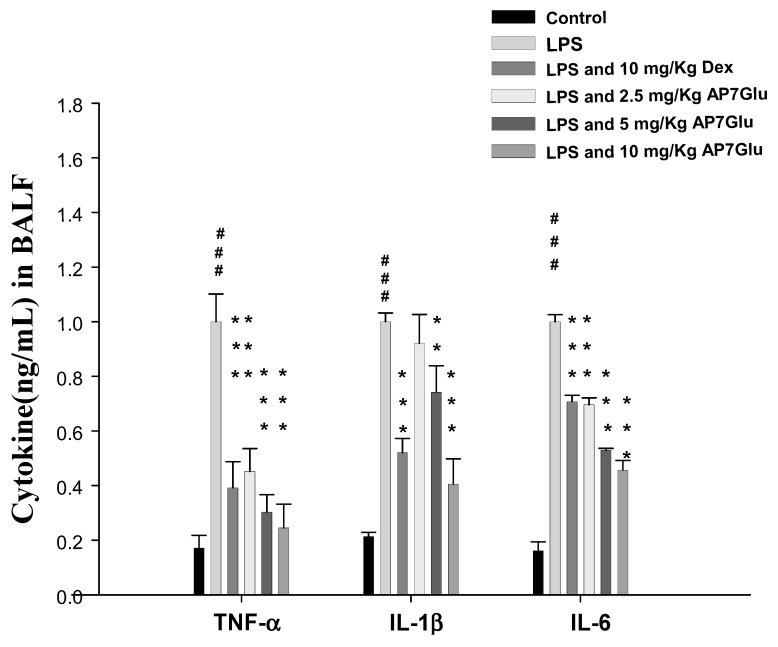
AP7Glu down regulated TNF-α, IL-6, and IL-1β in BALF. Six hours after LPS injection with or without AP7Glu pre-treatments, mice were sacrificed, their lungs were lavaged and the BALF were collected. TNF-α, IL-6 and IL-1β were detected by ELISA. Data represents mean ± SD of 6 mice. ^###^ compared with sample of control group. (One-way ANOVA followed by Scheffe’s multiple range tests). ** *p* < 0.01, and *** *p* < 0.001, were compared with LPS-alone group.

Pro-inflammatory cytokines, like TNF-α, IL-6, and IL-1β, in bronchoalveolar lavage (BAL) fluid were measured by ELISA 6 h after the insult. In Figure 5, the concentrations of cytokine in these groups of TNF-α, IL-6 and IL-1β in BALF of mice treated with LPS are seen only with the highest figures individually. However, the responses were inhibited by AP7Glu in a concentration dependent manner. More specifically, the sample with a higher concentration of AP7Glu showed the stronger inhibition ability for pro-inflammatory cytokines. Additionally, Dex also can decline the concentration of the pro-inflammatory cytokines in BALF. Finally, the above results have demonstrated that AP7Glu reduces the expression of pro-inflammatory cytokines, which in turn improves lung damage caused by LPS-induced ALI.

### 2.6. Effects of AP7Glu on MPO Activity and Antioxidative Enzymes in LPS Induced ALI

**Figure 6:**
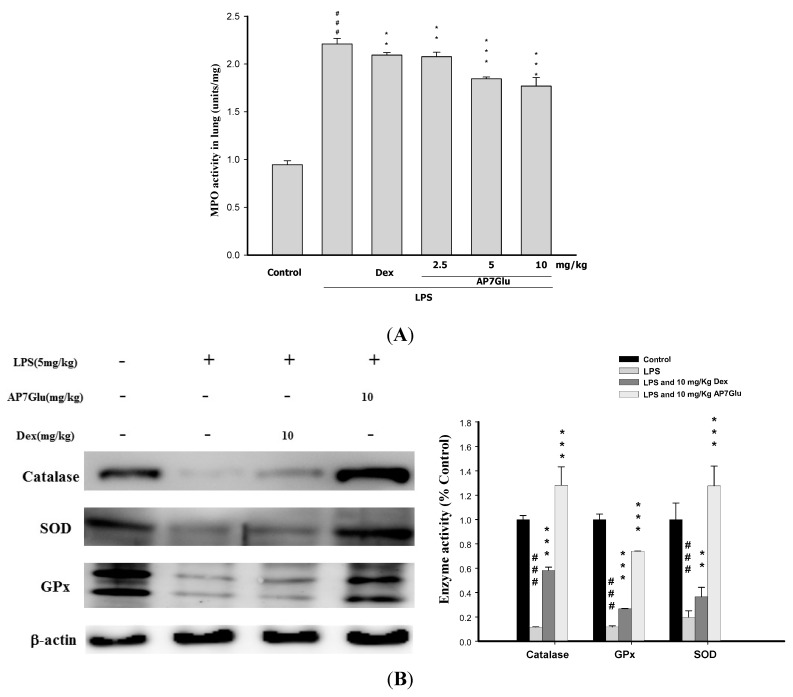
AP7Glu reduced (**A**) Myeloperoxidase activity (MPO) *in vivo*; (**B**) Antioxidative enzyme activation presented in western blotting. The antioxidative enzymes are represented SOD, catalase, and GPx which were performed at 6 h after LPS challenge. MPO activity was detected by ELISA reader described in Materials and Methods; its activity reflects the neutrophil infiltration in the lungs. Data represents mean ± SD of 6 mice. ^###^ compared with sample of control group. (One-way ANOVA followed by Scheffe’s multiple range tests). ** *p* < 0.01, and *** *p* < 0.001, were compared with LPS-alone group.

In other studies, activated PMNs have been linked to production of oxidative stress, which is recognized as a critical factor of pathophysiology of ALI. Moreover, there is a positive relation between the accumulation of PMNs and MPO activity, and AOEs, such as SOD, catalase, and GPx, are consumed during ALI. It means that AOEs can ameliorate inflammatory activities in LPS-treated mice [18,19]. According to our results in Figure 6A showing the level of lung MPO activity, all LPS groups are obviously increased after 6 h since the LPS challenge. At the same time, animals showed a marked increase of lung MPO activity (Figure 6A, *p* < 0.001), whereas animals pretreated with AP7Glu dose-dependently exhibited a trend toward lower levels. In Figure 6B, pretreatments with AP7Glu and Dex both activated SOD, catalase, and GPx, and the contents of these enzymes in LPS with AP7Glu groups are stunningly higher than that in the LPS alone group (Figure 6B, *p* < 0.001).

### 2.7. Inhibition of LPS Induced iNOs and COX-2 Proteins by AP7Glu in Lung Tissue

**Figure 7:**
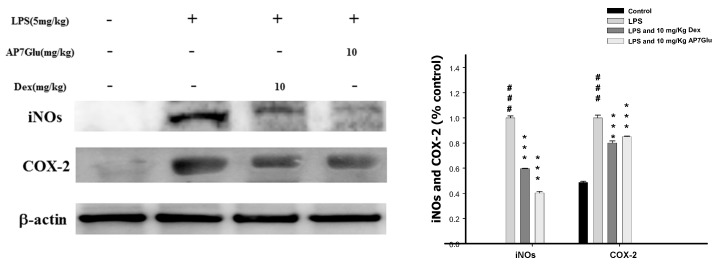
AP7Glu inhibited iNOs and COX-2 expression in lung. Seventy-two hours after LPS injection with or without AP7Glu pre-treatments, mice were exsanguinated and their lungs were removed. A representative Western blot from two separate experiments is shown and its relative protein levels were calculated with reference to a LPS-stimulated culture. Data represents mean ± SD of 6 mice. ^###^ compared with sample of control group. (One-way ANOVA followed by Scheffe’s multiple range tests). *** *p* < 0.001, were compared with LPS-alone group.

To assess the potential role of AP7Glu in LPS-induced ALI, we determined the level of cytokine proteins in the lung tissue of LPS-induced ALI mice using western blot. As shown in Figure 7, the levels of iNOs and COX-2 proteins in the AP7Glu + LPS group compared with the LPS group were significantly decreased (*p* < 0.001).

### 2.8. Effects of AP7Glu on MAPK, IκB and NF-κB Activation in LPS Induced ALI

**Figure 8:**
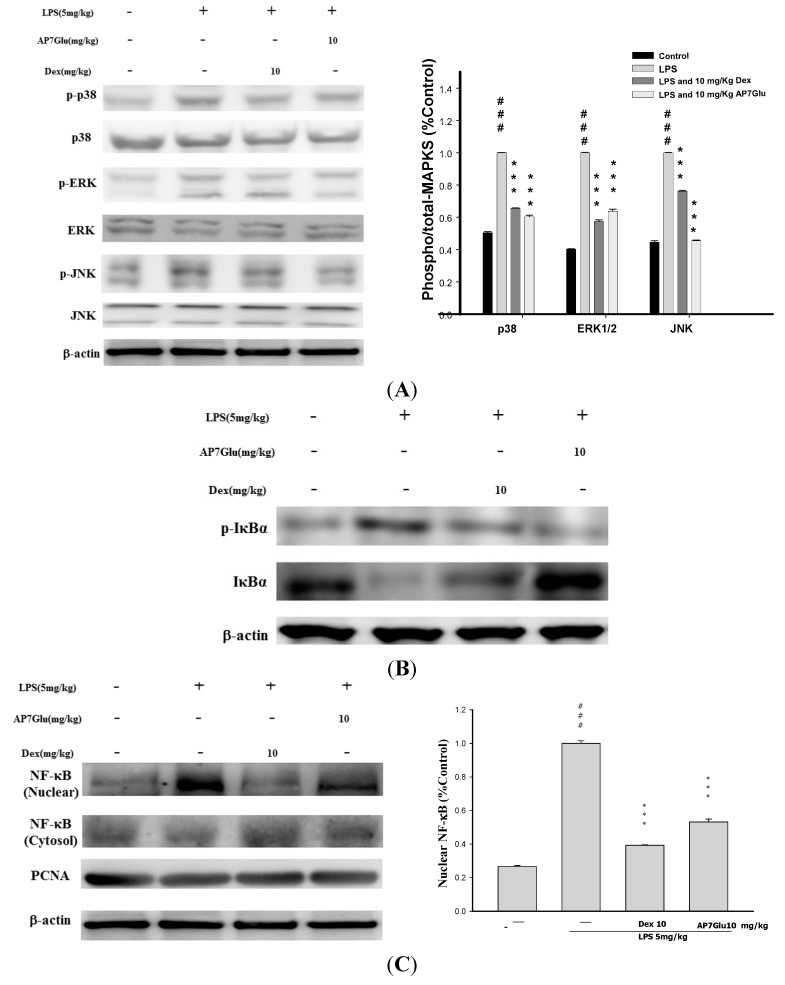
Effects of AP7Glu on LPS-induced (**A**) MAPK (**B**) IκBα, (**C**) NF-κB phosphorylation and non-phosphorylation protein expressions in ALI mice. Mice were pretreated with different concentrations of AP7Glu for 1 h and stimulated with LPS. Western blotting was performed using an antibody specific for the detection of IκBα phosphorylated, NF-κB nuclear and cytosol, and three forms of MAPK molecules, ERK, p38, and JNK. The fold change in protein expression between the treated and control groups was calculated. A representative Western blot from two separate experiments is shown. Data represents mean ± SD of six mice. ^###^ compared with sample of control group. (One-way ANOVA followed by Scheffe’s multiple range tests). *** and *** *p* < 0.001, were compared with LPS-alone group.

The three MAPK pathways, ERK, p38MAPK, and JNK have been demonstrated to participate in the activation of NF-κB in LPS-induced ALI [1,8]. The effects of AP7Glu on phosphorylation of ERK (on residue tyrosine-204), JNK (on residues threonine-183 and tyrosine-185), p38MAPK (on residues threonine-180 and tyrosine-182) and the associated protein pIκB in LPS-induced ALI were analyzed by Western blotting. The results showed that LPS stimulation significantly increased MAPK phosphorylation, and AP7Glu significantly inhibited LPS-induced phosphorylation of MAPK. Following the Figure 8A, it shows obviously that the percentages of phosphorylation in LPS with AP7Glu through the pathways of JNK and p38MAPK are lower than that in LPS with Dex at the same dose with 10 mg/kg. It also means that treating inflammation by AP7Glu is more effective than Dex through the pathways of JNK and p38MAPK.

In parallel with phosphorylation of MAPK, the effect of AP7Glu on IκB degradation was also investigated, whose effect is similar to the phosphorylation on MAPK, LPS administration induced an elevation in degradation of IκB; the increased degradation was significantly attenuated by AP7Glu. (*p* < 0.001; Figure 8B).

Phosphorylation of NF-κB prompts the transcription of most pro-inflammatory cytokines including TNF-α, IL-1β, and IL-6, and plays a pivotal role in the pathogenesis of ALI [9]. The effects of AP7Glu on phosphorylation of NF-κB p65 were measured by Western blotting. The serine phosphorylation of NF-κB p65 in lung increased significantly after LPS administration compared with the control group. Pretreatment with AP7Glu reduced phosphorylation of NF-κB p65 induction by LPS.

These data indicate that AP7Glu relieved LPS-induced ALI by inhibiting NF-κB activation through degradation of the p-NF-κB and p-IκB as well as JNK, ERK, and p38MAPK active phosphorylation pathways.

### 2.9. Effects of AP7Glu on HO-1 Expression in LPS-Induced ALI

**Figure 9:**
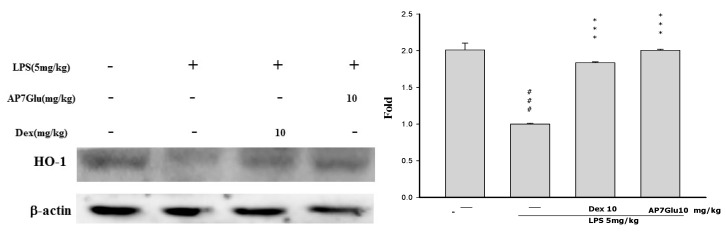
Effects of AP7Glu on LPS-induced HO-1 expression in lung. Tissue suspensions were prepared and subjected to Western blotting by using an antibody specific for HO-1, and β-actin was used as an internal control. The fold change in HO-1 expression between the treated and the control groups was calculated. Data represents mean ± SD of 6 mice. ^###^ compared with sample of control group. (One-way ANOVA followed by Scheffe’s multiple range tests). *** *p* < 0.001, were compared with LPS-alone group.

HO-1, is a kind of antioxidative protein to ameliorate symptoms of ALI through the inhibition of NF-κB phosphorylation [20]. During the experimental process of LPS-induced ALI, the effect of AP7Glu on HO-1 expression was analyzed by Western blotting. LPS stimulation exhibits two crucial results as shown in Figure 9: one is that there is a considerable increase in HO-1 expression, and another is that AP7Glu further enhances LPS-induced HO-1 expression in a concentration dependent manner. (*p* < 0.001). This suggests that AP7Glu with the antioxidative property of HO-1 has the ability to reduce LPS-induced oxidative stress.

## 3. Discussion

The present study clearly demonstrated AP7Glu with anti-inflammatory effect suppressed neutrophil infiltration in the lung and inhibited the expression levels of key pro-inflammatory cytokines such as TNF-α, IL-6, and IL-1β via two different routes of p-IκB and p-MAPK.

We found that AP7Glu had less cytotoxicity compared with apigenin, although both of them exhibited the similar effect on NO inhibition. (Figure S1A,B). Moreover, from our preliminary study, the mice were injected with apigenin and stimulated with LPS one hour after to induce inflammation for 6 h. At the end of experiment with a total of 7 h, the experimental results show that a large amount of yellow granules remained in the abdominal cavity of mice (unpublished data). The probable reason for this accumulation of apigenin in the abdominal cavity might be that apigenin was insoluble in the PBS. Therefore, AP7Glu, apigenin in glycosidic form, was chosen to use in this study because of its high solubility and NO inhibition function. In order to investigate whether the inhibition of NO production was due to decreased iNOs and COX-2 protein level, the effect of AP7Glu on iNOs and COX-2 protein expression was studied by immunoblot. The results showed that incubation with AP7Glu inhibited iNOs and COX-2 proteins expression in mouse macrophage RAW264.7 cells in a dose-dependent manner (Figure S2). The detection of b-actin was also performed in the same blot as an internal control.

Acute respiratory distress syndrome (ARDS) is a type of acute and progressive respiratory disease without the symptom of abnormal cardiac filling pressure. In fact, the obvious character is to diffuse bilateral pulmonary edema progressively, and inflammation. Moreover, it even reduces pulmonary compliance and hypoxemia [21,22]. Symptoms of ALI are induced by progression of different illnesses, with a similar pathophysiological process. ALI ensues irrespective of whether the damage directly occurs on the alveolar epithelial cells by an external stimulus or through an indirect process resulting from a more distant systemic inflammatory process mediated via cytokines. Both pulmonary inflammation and pulmonary edema are the most important pathological findings in ALI.

LPS is found in the outer membrane of Gram-negative bacteria. It also is one of the main pro-inflammatory reaction factors in infectious diseases, which lead to over inflammatory reactions *in vivo*. When LPS was intratracheally instilled, the main inflammatory cells, PMNs, were able to release enzymes and phagocytizing pathogens after infiltrating into lung tissues and BALF. Meanwhile, these inflammatory cells were the fundamental source of inflammatory mediators *in vivo*, including IL-6, IL-1β, and TNF-α. After activation, there are two results in LPS-induced ALI animal models, more specifically, for the former LPS group outcome, the total number of PMNs and cytokines in BALF were significantly increased. For the AP7Glu group outcome, AP7Glu reduced the production of TNF-α, IL-1β, and IL-6 and PMNs in BALF. Therefore, the results also indicated that AP7Glu reduced leukocyte infiltration in the lungs by decreasing the expression of pro-inflammatory cytokines (Figure 5).

According to above perspectives, AP7Glu has been shown to be a kind of endogenous factor. These findings suggest this flavone could be a potential alternative for the treatment of immunological disorders [23]. In response to LPS, it is well known that the mechanism of LPS inducing NF-κB is a crucial factor of endogenous transcription to modulate inflammation. At the beginning, in unstimulated cells, inactivated NF-κB dimers are sequestered in the cytosol via covalent interactions with IκB as a kind of an inhibitor protein. After phosphorylation stimulation, p-IκB causes the release of NF-κB, allowing the p65 subunit to process rapid phosphorylation and then translocate into the nucleus [24,25]. We found that pretreatment with AP7Glu could prevent both activation of NF-κB p65 and phosphorylation of IκB in the lungs of LPS-induced ALI (Figure 8B,C). In addition, the present study demonstrates that AP7Glu reacted with intratracheal instillation of LPS in mice resulting in phosphorylation of ERK, p38MAPK, and JNK in lung tissue [26,27]. Hence, that also meant that AP7Glu pretreatment was associated with NF-κB p65 activation pipeline in in lung through activation of MAPK and IκB.

With regard to MAPK, it also plays an important role in PMN activation, such as transmigration, degranulation, and respiratory burst [28]. During the respiratory burst, the amount of oxygen consumption is converted into superoxide anions through nicotinamide adenine dinucleotide phosphate oxidase, which would have a ripple effect in the body to destroy surrounding cells. Nevertheless, such effect could be eliminated by SOD which exists in the cytoplasm, because SOD can catalyze the reduction of superoxide anions into oxygen and hydrogen peroxide.

Furthermore, under normal physiological conditions, oxidative damage is improved by antioxidative enzymes that are acquired through SOD, catalase, and GPx. Notably, superoxide anions related to oxidative damage are converted into hydrogen peroxide by SOD, which is then metabolized to water by catalase or GPx [1,29]. Flavone has been shown previously to increase the levels of SOD and catalase in murine models [30,31]. In this study, we demonstrated that pretreatment with AP7Glu raised the activation levels of SOD, catalase, and GPx in LPS-induced ALI (Figure 6B). These observations suggest that AP7Glu is capable of reducing serious lung damage through AOEs.

In terms of MPO as a detector, its release via PMN degranulation can catalyze hydrogen peroxide that induces the formation of TNF-α, IL-6, and IL-1β. Not only these induced compounds, but also overproduction of NO from iNOs has been implicated as an important mediator in the pathogenesis of inflammation. However, the inhibition of NO production involvement in the anti-inflammatory activity of AP7Glu is not known. We assessed the anti-inflammatory effects of AP7Glu through proposing a novel reaction mechanism to demonstrate that AP7Glu significantly inhibited induction of LPS-induced NO production in macrophages, which may be associated with the attenuation of activation for NF-κB, pro-inflammatory cytokines, and MPO. Our results also suggested that IL-1β, IL-6, and TNF-α were direct targets of reaction for AP7Glu and suppressed downstream signaling pathways to decrease iNOs and COX-2 protein expressions (Figure 5 and Figure 7). A previous study reported that a pharmacological NF-κB inhibitor reduced inflammatory iNOs expression [9,32], which is in parallel with our results.

Heat shock protein32, also called HO-1, is a type of inducible defense enzyme against oxidative stress. Moreover, data from various studies have reported that increased HO-1 expression plays a vital role in the inhibitory modulation of not only NF-κB expression, but also NO production in LPS-activated macrophages [17,33]. Therefore, HO-1 may be a target for the treatment of inflammatory disorders. A previous study has suggested that a marker of activation HO-1 could be a signal injury occurring in LPS-induced ALI, such as lung permeability and generation of IL-6 [34]. The above perspective is in agreement with the result demonstrated from our data that AP7Glu prompted expression of HO-1 in LPS-induced ALI (Figure 9).

## 4. Experimental Section

### 4.1. Cell Culture

A murine macrophage cell line RAW264.7 (BCRC No. 60001) was purchased from the Bioresources Collection and Research Center (BCRC) of the Food Industry Research and Development Institute (Hsinchu, Taiwan). Cells were cultured in plastic dishes containing Dulbecco’s Modified Eagle Medium (DMEM, Sigma, St. Louis, MO, USA) supplemented with 10% fetal bovine serum (FBS, Sigma, USA) in a CO_2_ incubator (5% CO_2_ in air) at 37 °C and subculture every other day at a dilution of 1:5 using 0.05% trypsin 0.02% EDTA in Ca^2+^, Mg^2+^ free phosphate-buffered saline (DPBS).

### 4.2. Cytotoxicity and NO Production

This preliminary experiment consists of two parts. In the first part, we examined the viability of RAW cells; as for the second portion, the cell medium was examined for NO production.

RAW 264.7 cells (3 × 10^4^ per well) were seeded in 96-well plates containing DMEM supplemented with 10% FBS for 1 day to become nearly confluent. On the second day, the cells were pre-treated with the indicated concentrations of AP7Glu 1 h before treatment with LPS (100 ng/mL) at 37 °C for 24 h. The media were removed and stored for the following NO experiment, while the cells were incubated with 100 μL of 0.5 mg/mL MTT in a humidified atmosphere of 5% CO_2_ and 95% air for 6 h at 37 °C. The medium was discarded, followed by the addition of 100 μL isopropanol. After incubation for 30 min, the absorbance was read using a microplate reader at 570 nm. The above steps were repeated three times for each concentration.

The nitrite level in cultured media, which reflects intracellular NO synthase activity, was determined by Griess reaction [35]. 100 μL of Griess reagent (1% sulfanilamide, 0.1% naphthyl ethylenediamine dihydrochloride and 5% phosphoric acid) was added to each sample medium and incubated at room temperature for 10 min. By using sodium nitrite to generate a standard curve, the concentration of nitrite for each sample was measured at the absorbance of 540 nm.

### 4.3. Animals

Seventy two male Institute for Cancer Research (ICR) mice, 6 weeks old, were obtained from BioLASCO Co., Ltd., Taipei, Taiwan. The animals were kept in plexiglass cages at a constant temperature of 22 ± 1 °C, relative humidity 55% ± 5% and with 12 h dark-light cycles. They were given food and water *ad libitum*. Animal studies were conducted according to the regulations of Instituted Animal Ethics Committee, and the protocol was approved by the Committee for the Purpose of Control and Supervision of Experiments on Animals. Mice were randomly divided into six groups of animals (*n* = 12). Mice in the normal control and negative control groups were given distilled water *ad libitum*.

### 4.4. Model of LPS Induced ALI

Seventy-two male ICR mice were randomly divided into 6 groups (*n* = 12): control group, LPS group, dexamethasone (DEX) group (10 mg/kg), low dosage AP7Glu group (2.5 mg/kg) (Sigma, St. Louis, MO, USA), middle dosage group (5 mg/kg, LPS + AP7Glu-M) and high dosage group (10 mg/kg, LPS + AP7Glu-H). Half of each group was taken for the inflammation protein analysis, slicing, and edema and the rest used for BALF analysis. ALI was induced by LPS (*E. coli* LPS serotype O55:B5, Sigma, St. Louis, MO, USA) via intratracheal injection [36]. In brief, mice were anesthetized with mixed reagent of 10 μL/g i.p., urethane (0.6 g/mL) and chloral hydrate (0.4 g/mL), followed by DEX (10 mg/kg) or AP7Glu intraperitoneal injection with individual doses. One hour after, LPS was given at 5 mg/kg in sterile saline intratracheal injections in 50 μL. The mice in the control group were administrated with sterile saline instead. The mice were then placed in a vertical position and rotated for 1 min to distribute the instillation in the lungs. Six hours later, the mice were sacrificed.

### 4.5. Bronchoalveolar Lavage Fluid (BALF), Total Cell Count and Protein Analysis

Six hours later, mice were exsanguinated after anesthesia. According to the previous report, BALF was collected at the upper part of the trachea, by lavage three times with 500 μL PBS (pH 7.2) each time. The fluid recovery rate was more than 90%. Lavage samples from mice were kept on ice. BALF was centrifuged at 700× *g* for 5 min at 4 °C [36]. The supernatant was removed and retained. The sedimented cells were resuspended in 2 mL PBS, of which 1 mL was used to detect cell counts by cytometer and the other to detect total protein content which with a RIPA solution (radioimmuno-precipitation assay buffer) and centrifuged again to obtain the supernatant in order to detect total protein content by Bradford assay.

### 4.6. TNF-α, IL-6, and IL-1β Cytokines in BALF

The BALF supernatant was collected after centrifugation (for 5 min at 700× *g*) and stored at −80 °C before the cytokine assay. TNF-α, IL-6 and IL-1β in BALF were measured by an enzyme-linked immunosorbent assay ELISA R&D Systems (Minneapolis, MN, USA) by modifying a previously reported method [37]. The limit of detection of this method was greater than 7.8 pg/mL.

### 4.7. Myeloperoxidase (MPO) Activity Assay

After BALF collection, the left upper lobe was removed, washed and kept in −80 °C. The steps were conducted according to the method of Bani et al. [38] with some modifications. After weighing, the lungs were homogenized at 12,000× *g* and 4 °C for 15 min and resuspended in 50 mM KPO_4_ buffer (PH 6.0) with containing 0.19 mg/mL of o-dianisidine chloride and 0.0005% H_2_O_2_ was a substrate for myeloperoxidase at 460 nm with a spectrophotometer (Molecular Devices, Sunnyvale, CA, USA). The results were expressed as units of MPO activity per gram of lung tissue.

### 4.8. Lung Wet/Dry Weight Ratio

The severity of pulmonary edema was assessed by the wet to dry ratio (W/D ratio). The right lower lungs were weighed and then dehydrated at 60 °C for 72 h in an oven [39].

### 4.9. H&E Staining

The left lower lung from each mouse was fixed in 10% formalin, embedded in paraffin, cut into 5 mm sections before being stained with H&E. Lung injury score was measured by a blinded pathologist with a 0 to 5 point scale according to combined assessments of inflammatory cell infiltration in the airspace or vessel wall, alveolar congestion, hemorrhage, alveolar wall thickness and hyaline membrane formation. A score of 0 represented no damage; l represented mild damage; 2 represented moderate damage; 3 represented severe damage; and 4–5 represented very severe histopathological changes [40].

### 4.10. Western Blot Analysis of Lung Tissue

PBS and RIPA were added to lung tissue before grinding. The extract was then centrifuged at 12,000× *g* for 15 min to obtain the supernatant. Bovine serum albumin (BSA) was used as a protein standard to calculate the equal total cellular protein amounts. Protein samples (50 μg) were resolved by denaturing 10% sodium dodecyl sulfate-polyacrylamide gel electrophoresis (SDS-PAGE) using standard methods, and then were transferred to PVDF membranes by electroblotting and blocking with 1% BSA. The membranes were probed with primary antibodies (iNOs, COX-2) for phosphorylated and non-phosphorylated forms of p38 MAPK, ERK, IκB-α, JNK, HO-1, p65 and pro-inflammatory enzymes (SOD, GPx, catalase) at 4 °C overnight, washed three times with PBST, and incubated for 1 h at 37 °C with horseradish peroxidase conjugated secondary antibodies. The membranes were washed three times before being detected for immunoreactive proteins with enhanced chemiluminescence (ECL) using hyperfilm and ECL reagent. The results of Western blot analysis were quantified by measuring the relative intensity compared to the control by using Kodak Molecular Imaging Software and represented in relative intensities.

### 4.11. Statistical Analysis

Unless otherwise stated, all experiments were performed at least three times independently. The data were presented as the means ± SD and statistical comparisons between the groups were performed using one-way ANOVA, followed by a Scheffe’s multiple range test. A *p*-value less than 0.05 were considered significant.

## 5. Conclusions

The present study reported the anti-inflammatory effect of apigenin-7-glycoside in LPS-stimulated acute lung injury and related detailed the molecular mechanisms for the first time. We consider that AP7Glu is an important natural product with few side effects and low toxicity; therefore AP7Glu may be a promising therapeutic candidate for various lung inflammatory disorders, such as lung disease and obstructive pulmonary.

## Supplementary Materials

Supplementary materials can be found at http://www.mdpi.com/1422-0067/16/01/1736/s1.
